# Short term hemodynamic effects of atrial fibrillation in a closed-loop human cardiac-baroreflex system

**DOI:** 10.1371/journal.pone.0334086

**Published:** 2025-10-29

**Authors:** Oluwasanmi Adeodu, Michelle Gee, Babak Mahmoudi, Rajanikanth Vadigepalli, Mayuresh V. Kothare

**Affiliations:** 1 Department of Chemical and Biomolecular Engineering, Lehigh University, Bethlehem, Pennsylvania, United States of America; 2 Department of Chemical and Biomolecular Engineering, University of Delaware, Newark, Delaware, United States of America; 3 Department of Biomedical Informatics and Biomedical Engineering, Emory University, Atlanta, Georgia, United States of America; 4 Department of Pathology and Genomic Medicine, Thomas Jefferson University, Philadelphia, Pennsylvania, United States of America; Chung-Shan Medical University Hospital, TAIWAN

## Abstract

Atrial fibrillation (AF) remains the leading cardiac cause of stroke and AF-related death rate in the United States has been increasing for over twenty years. While the effect of standalone AF on heart rate is well established, there is a lack of clarity on its impact on other critical hemodynamic metrics. This is ostensibly due to interaction with other common comorbidities, especially hypertension. In addition, AF has a complex relationship with the state of the baroreflex. Evidence indicates that baroreflex sensitivity (BRS), the ability of the intrinsic cardiac control system to initiate parasympathetic response, is suppressed during AF. Therefore, a proper assessment of the hemodynamic impact of AF must take the state of the baroreflex into consideration. In this paper, we present a lumped parameter model of the human cardiovascular-baroreflex system that adequately translates AF-induced electrophysiological changes to measurable hemodynamic effects. We consider the stochastic effects of the electrical disruption in the sinus node, the absence of atrial contraction and BRS suppression. Our model provides insight into the impact of standalone AF on key benchmarks: heart rate, arterial pressure and stroke volume, under varying degrees of BRS suppression. In addition, the development of a tractable mathematical model is essential for the in-silico evaluation of emerging neuromodulation therapies for AF. Our model predictions are in agreement with published clinical data and suggest that high blood pressure during standalone AF is strongly dependent on the extent of damage to the baroreflex, which may explain conflicting reports of AF-related hypertension and normotension.

## Introduction

**Fig 1:**
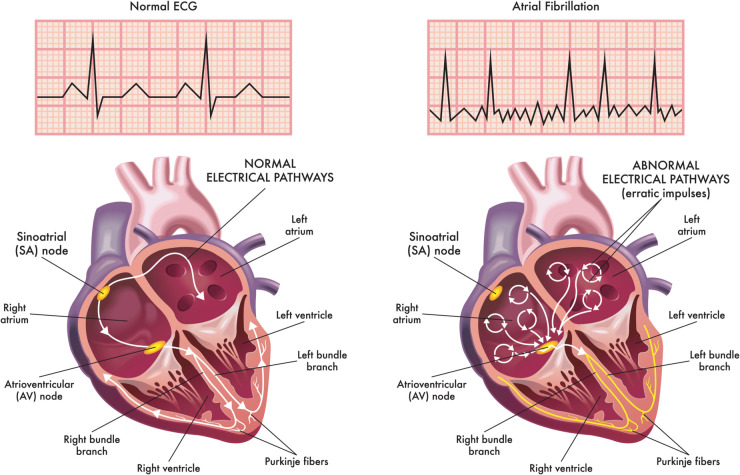
Electrical activity within the heart chambers during normal sinus rhythm and AF. Propagation of electrical activity in the sinus and atrioventricular nodes (bottom) and representative electrocardiograms (ECG) (top). Illustration by rob3000/Adobe Stock

Atrial fibrillation (AF) is the most common type of arrhythmia and the leading cardiac cause of stroke [1], directly contributing to over a hundred thousand deaths each year. It is projected that by 2050, AF will affect between 6 to 12 million people in the United States [2]. The pathology of AF is characterized by an abnormal, high frequency excitation of the atria (Fig 1) that results in dyssynchrony between the upper and lower chambers of the heart. The loss of atrioventricular coordination reduces the effectiveness of ventricular filling and consequently, the volume of blood in systemic circulation, causing dizziness or syncope. In addition, an ineffective left atrial (LA) discharge into the left ventricle (LV) can lead to an accumulation of blood in the LA, increasing the risk of coagulation and stroke.

AF is typically classified based on the duration (or severity) of the fibrillation event: paroxysmal (less than seven days), persistent (more than seven days) or permanent. Current treatment for milder forms of AF involves beta-blockers (for rate control) and anticoagulants. However for drug refractory AF, cardioversion or ablation of the malfunctioning myocytes may be necessary to restore a normal sinus rhythm. However, the rate of AF recurrence after a cardioversion or ablation procedure can be as high as 40% [3,4]. Moreover, the inadvertent transection of adjacent ganglionated plexi during pulmonary vein ablation can cause vagal denervation and increase the likelihood of AF recurrence [5,6].

Clinical evidence indicates that the genesis and maintenance of AF is intricately linked with dysfunction of the cardiac autonomic system [6,7]. An increase in vagal activity has been identified as a significant precursor of paroxysmal AF episodes [6] while persistent AF seems to be induced by excessive sympathetic activity [8]. On the other hand, the maintenance of AF is characterized by the withdrawal of parasympathetic tone as evidenced by the reduction of baroreflex sensitivity (BRS) during AF episodes [9]. These observations show that a proper study of the impact of AF must account for the baroreflex.

Previous efforts to develop mechanistic models of AF (for example, [10–13]) focus on providing insight into the generation and continuation of AF. Typically, these multi-scale models incorporate electrical activity using cellular ionic models and three-dimensional representations of the atria obtained from imaging techniques, and provide simulations of the propagation of errant wavelets linked to the initiation and propagation of AF in the sinus node. Although these computational models have promoted the understanding of the underlying mechanisms that determine AF predilection and increased the potential of simulation-guided ablation [14], they neglect a broad but clinically-relevant overview: the net effect of AF on measurable hemodynamic metrics. Moreover, these models are represented by computationally expensive systems of partial differential equations and are therefore not suited for the rapid in-silico testing of emerging neuromodulation therapies to assuage or even reverse AF [15].

In contrast, our goal is to develop a lumped parameter model of the human cardiovascular-baroreflex system that translates AF-induced electrophysiological phenomena to their easier-to-measure effect on hemodynamic variables. Therefore, we focus on a global representation of errant electrical activity and neglect spatial variations within cardiovascular compartments. We demonstrate that this lumped parameter approach sufficiently captures the target phenomena and ensures the model is a tractable system of ordinary differential equations. The computational AF model by Scarsoglio *et al*. [16] employed a similar lumped parameter approach in their description of the cardiovascular system but they failed to account for the important effect of baroreflex suppression during AF. To the best of our knowledge, the work we present is the first computational study of the hemodynamic effects of AF that accounts for the state (healthy or impaired) of the baroreflex.

**Fig 2:**
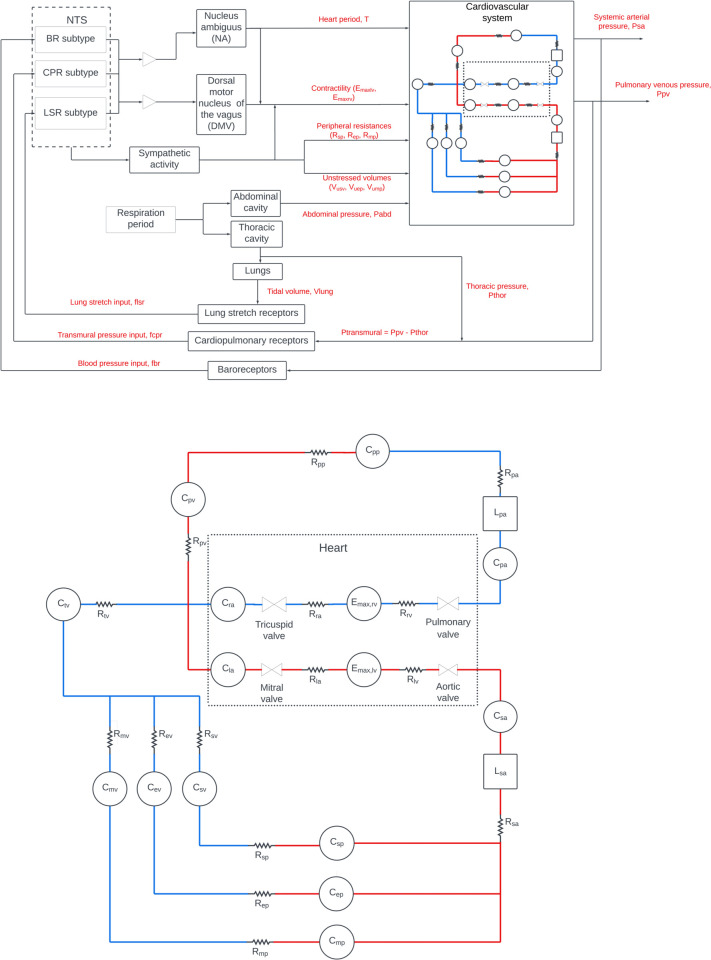
A model of the human cardio-baroreflex system. Top: Information flow of the autonomic regulation of the cardiovascular system from Park *et al*. [17]. Bottom: A hydraulic analog of the systemic and pulmonary circulatory systems. Red, oxygenated blood; blue, de-oxygenated blood; *P*, pressures; *R*, hydraulic resistances; *C*, compliances; *L*, inertances; *sa*, systemic arteries; *sp*, splanchnic peripheral circulation, *sv*, splanchnic venous circulation; *ep*, extrasplanchnic peripheral circulation, *ev*, extrasplanchnic venous circulation; *mp*, muscular peripheral circulation, *mv*, muscular venous circulation; *ra*, right atrium; *rv*, right ventricle; *pa*, pulmonary arteries; *pp*, pulmonary peripheral circulation; *pv*, pulmonary veins; *la*, left atrium; *lv*, left ventricle

The ensuing sections are divided as follows. We start with a validated model of the human cardio-baroreflex system from Park *et al*. [17] depicted in Fig 2. In the next section, we upgrade Park *et al*.’s description of left atrial operation to a more realistic version by re-defining the LA as a pulsating unit and accounting for differences in LA elasticity during contraction and expansion. Thus, we establish a new baseline during normal sinus rhythm. Then, in order to capture short term physiological changes associated with the onset of AF, we introduce three modifications to the model namely, (1) the disappearance of atrial contraction, (2) the propagation of errant stimulation through the atrioventricular node, causing irregular, heightened heart rates and (3), the suppression of baroreflex sensitivity. Simulations of various hemodynamic quantities during normal sinus rhythm at rest, sensitivity analysis and comparisons with clinical data of various hemodynamic quantities are presented in the Results section.

## A new baseline: The pulsating left atrium

**Fig 3:**
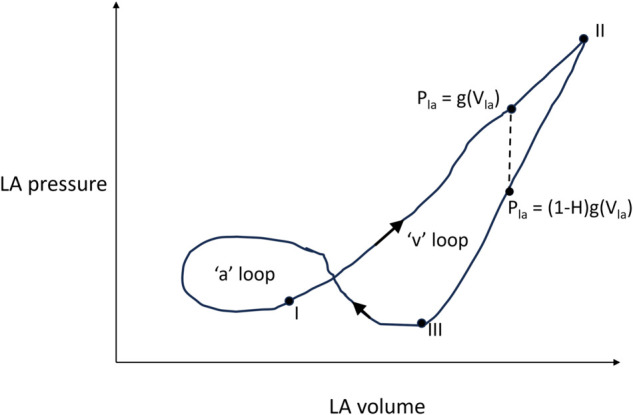
A sketch of the left atrial (LA) pressure-volume relationship within a cardiac cycle. The ‘v’ and ‘a’ loops are associated with passive emptying and atrial contraction respectively.

In the model of Park *et al*. [17], the pressure-volume relationships in non-ventricular compartments of the cardiovascular system Fig 2 were defined to be simple linear functions with constant elastance gradients. Details of these equations and parameter values are provided in the supplementary text S1 File. However, since AF involves a drastic change in left atrial (LA) function, it is essential to capture the more subtle aspects of baseline LA dynamics. As depicted in Fig 3, the LA pressure-volume relationship over the course of a complete heart cycle has a characteristic double-loop shape.

Starting from the onset of ventricular systole marked ‘I’ in Fig 3, the segment I-II represents LA reservoir function when it accumulates blood from the pulmonary veins. Section II-III represents the conduit phase where blood from the LA empties passively into the left ventricle (LV) as a result of a significant transmitral pressure difference. It is crucial to note that LA filling and emptying follow separate pressure-volume pathways, creating the ‘v’ loop. The area of the ‘v’ loop is regarded as a metric of reservoir function [18,19] but in mechanical terms, it is simply the difference between the energy absorbed in the reservoir phase and expended during the conduit phase. That is, the ‘v’ loop area can be regarded as a measure of elastic hysteresis. Transmitral flow diminishes as pressures in the two chambers equalize, marking the start of diastasis. Then, to complete the cycle, atrial contraction or kick causes a rapid spike in LA pressure causing an anticlockwise pressure-volume trajectory (segment III-I) and creating the ‘a’ loop such that its area is a measure of the work done during contraction.

Very few lumped parameter models of the cardiovascular system predict a realistic operation of the LA with reservoir-conduit hysteresis and atrial contraction. Pironet *et al*. [20] proposed a multi-scale model that links the variations of intracellular calcium within the LA and LV to the forces exerted on their walls, and ultimately to volume and pressure changes. However, this model required fitting twelve parameters to adequately reproduce the hemodynamics of a typical canine. Noreen *et al*. [21] proposed a piecewise approach to LA modeling by distinguishing between LA elastic properties during contraction and expansion. However, a framework for the selection of suitable parameters was not provided. We adopt a framework similar to [21] but employ a compact, intuitive description of hysteresis to reduce the number of free variables in the subsequent parameter assignment step.

Clinical recordings of the PV relationship of the LA during filling (${\dot{V}}_{la}\geq 0$) are well described by exponential functions [22,23] of the form

$$P_{la}=b\cdot e^{a(V_{la}-V_{u,la})}$$ (1)

where *a* and $V_{u,la}$ are the LA elastic stiffness constant (ml^−1^) and unstressed volume (ml) respectively, *b* is the baseline LA pressure in its unstressed state. However, the description of the PV relationship during the passive emptying and diastasis stages is more complex due to the simultaneous LA-LV interaction through the open mitral valve and the upstream influence of the pulmonary veins. Instead, we define a simple volume-dependent hysteresis metric, *H*, to quantify the pressure drop relative to the reservoir stage as shown in Fig 3. *H* varies with $V_{la}$ such that there is no hysteresis at the end-systolic, $V_{la,es}$ and unstressed, $V_{u,la}$, volumes of the atrium

$$H=H_{max}\cdot \sin\left(\pi \cdot \frac{V_{la}-V_{u,la}}{V_{la,es}-V_{u,la}}\right)$$ (2)

and *H*_*max*_ is the maximum value of *H* at the mid-point between $V_{la,es}$ and $V_{u,la}$. We ascribe atrial contraction to a time varying atrial elastance that is governed by an atrial activation function, ϕ(t), such that contraction elastance, *E*_*la*_ rises from zero to a peak, *E*_*max*,*la*_ midway through contraction.

$$E_{la}(t)=E_{max,la}\cdot \phi (t)$$ (3)

It should be noted that atrial and ventricular contractions overlap. For the contraction activation function, we employ a modified form of the half sine function used for ventricular activation in [24] such that peak atrial contraction occurs at end of the ventricular cycle:

$$\phi (t)=\left\{ \begin{array}{cc} 0, & \;0\leq u\leq \frac{T-T_{kick}}{T},\\ \sin^{2}\left[\frac{\pi \cdot \left(uT-T+T_{kick}\right)}{T_{kick}}\right], & \;\frac{T-T_{kick}}{T}<u\leq 1 \end{array}\right.$$ (4)

where u∈[0,1] is the elapsed time in a cardiac cycle, with *u* = 0 marking the start of ventricular contraction. *T* is the length of the cardiac cycle and *T*_*kick*_ is the the duration of LA contraction and is estimated from P-R interval on ECG recordings. We use the empirical relationship [25] that was shown to be valid over a wide range of heart periods.

$$T_{kick}\text{(s)}=\frac{0.021}{T\text{(s)}}+0.1767$$ (5)

The left atrial PV relationship during a cardiac cycle can be summarized as

$$P_{la}(t)=\left\{ \begin{array}{cc} \left(1-\phi \right)\cdot be^{a(V_{la}-V_{u,la})}+E_{la}\left(V_{la}-V_{u,la}\right), & {\dot{V}}_{la}\geq 0\\ \left(1-\phi \right)\cdot \left(1-H\right)be^{a(V_{la}-V_{u,la})}+E_{la}\left(V_{la}-V_{u,la}\right), & {\dot{V}}_{la}<0 \end{array}\right.$$ (6)

The substitution of Eq 6 in place of the reductive linear PV relationship in the dynamical model of the human cardio-baroreflex system [17] constitutes a significant model upgrade. The improved model can be expressed in discrete state space notation as

$$x_{i+1}=f(x_{i},p)$$ (7)

$$y_{i}=g(x_{i},p)$$ (8)

where *i* is the heart cycle index; the vector of states, *x*, comprises of the blood volumes of the various compartments in the cardiovascular system, splanchnic and extrasplanchnic resistances, sympathetic and vagal contributions to heart rate and ventricular contractility; the output vector *y* consists of hemodynamic variables of interest such as heart rate, mean arterial pressure and stroke volume. Thus, *f* and *g* (provided in the supplementary text S1 File) describe the system of ordinary differential equations (or equivalently, in the form of forward difference equations of the heart cycle index) that capture the temporal evolution of the states. The vector *p* comprises of tunable parameters within the pulmonary circulation system, including the newly introduced quantities *a*, *b*, and *H*_*max*_. Then, the parameter tuning task to ensure that hemodynamic variables under rest conditions lie within known physiological ranges is the following optimization problem

$$\min_{p}c^{T}\Delta y\text{ s.t.}$$ (9)

$$x_{i}=f(x_{i},p)$$ (10)

$$y_{i}=g(x_{i},p)$$ (11)

$$y_{i}\in Y.$$ (12)

The constraint of Eq 12 specifies that each hemodynamic output of the dynamical system must lie within an acceptable physiological range, Δy is the deviation from known set points within *Y*, and *c* is the vector of penalties associated with each deviation. Eq 12 constraints includes the following

1. Under rest conditions, atrial kick contributes about 20% of stroke volume. [18].
2. The area of the ‘a’ loop is approximately 8 times the area of the ‘v’ loop [23] i.e.
  $$\iint_{\text{a loop}}\;dA≊8\iint_{\text{v loop}}\;dA$$ (13)
3. Mean arterial pressure is 93mmHg.
4. Average heart rate is 70 beats per minute.

We adopt the same numerical search strategy employed in [17] to solve Eq 9. A bounded space for the search was constructed by limiting admissible parameters to within 25 - 100% of values found in literature. Then, candidate combinations generated from Sobol sampling [26] of the constrained parameter space were used to simulate steady-state cardiovascular behavior to determine the optimal combination during sinus rhythm, *p*^(*SR*)^.

## AF-specific modifications to the baseline model

### Changes in the left heart

The over-stimulation of the atria by abnormal electrical impulses during AF are observable on an electrocardiogram (ECG). During AF, ‘P’ waves that represent the depolarization of the atria are absent. Instead, irregular, low amplitude ‘F’ waves precede the QRS complex. In other words, full atrial contractions are reduced to rapid, irregular spasms such that the contribution of atrial contraction to ventricular filling is non-existent. Accordingly, the atrial contraction function, *ϕ* in Eq 6, is set to zero. In addition, LA distention and a resultant fibrosis of the atrial muscle has been observed in patients with chronic AF [27]. We capture this potential engorgement and stiffening of the LA by defining a new unstressed LA volume $V_{u,la}^{\left(AF\right)}$ and passive elastic constant, *a*^(*AF*)^ such that


$$V_{u,la}^{\left(AF\right)}\geq V_{u,la}^{\left(SR\right)}$$

$$a^{\left(AF\right)}\geq a^{\left(SR\right)}$$

Thus, the PV relationship in the left atrium during AF is described by

$$P_{la}(t)=\left\{ \begin{array}{cc} be^{a^{\left(AF\right)}(V_{la}-V_{u,la}^{\left(AF\right)})}, & {\dot{V}}_{la}\geq 0\\ \left(1-H\right)be^{a^{\left(AF\right)}(V_{la}-V_{u,la}^{\left(AF\right)})}, & {\dot{V}}_{la}<0 \end{array}\right.$$ (14)

Perhaps the most well-known effect of AF is the ‘irregularly irregular’ R-R interval (or heart period, *T*). The atrioventricular (AV) node, also known as the heart’s secondary pacemaker, filters the conduction of errant impulses from the atria to the ventricles. However, this filtering function is compromised during AF. Thus, the effect of the chaotic signals on ventricular beats and ultimately on systemic blood circulation is dependent on the properties or state of the AV node. Various studies, (for example, [28–32]) have been conducted on the statistical properties of R-R intervals during AF episodes to provide insight into the underlying pathophysiology or to develop AF detection algorithms. These studies generally agree that the autocorrelation, if any, in a sequence of R-R intervals during AF is not significant beyond a few heartbeats. Therefore, for modeling purposes, each heart period may be randomly selected from a pre-defined distribution. While the atrial activation interval distribution during AF is generally modeled as a Gaussian, clinical instances of bimodal and multi-modal R-R interval distributions are not uncommon [28,33].

We adopt the theoretical approach proposed by Zeng and Glass [34] to describe the filtering effect of the AV node during AF. In their work, [34], the R-R distribution is modeled as a function of the interval between successive atrial activations (A-A), the refractory period of the AV-node, *θ*, and the atrioventricular recovery function, *h*, that links ventricular recovery time (V-A interval) to the conduction time (A-V interval) through the AV-node. The recovery curve is described as a double exponential function to account for fast and slow conduction pathways through the AV node. The existence of two conduction pathways through the AV-node, referred to as the dual AV-node physiology, is intricately linked to tachycardic arrhythmia. Indeed, bimodal R-R interval distributions have been interpreted as evidence of dual AV node physiology and can serve as a predictor of the efficacy of radiofrequency catheter modification of the AV node [33]. We express Zeng and Glass’ [34] mathematical model in recursive form and drop the hyphens for ease of notation:

$$AV_{k+1}=h(VA_{k})=AV_{min}+\alpha_{1}\cdot e^{\frac{-VA_{k}}{\tau_{1}}}+\alpha_{2}\cdot e^{\frac{-VA_{k}}{\tau_{2}}}.$$ (15)

**Fig 4:**
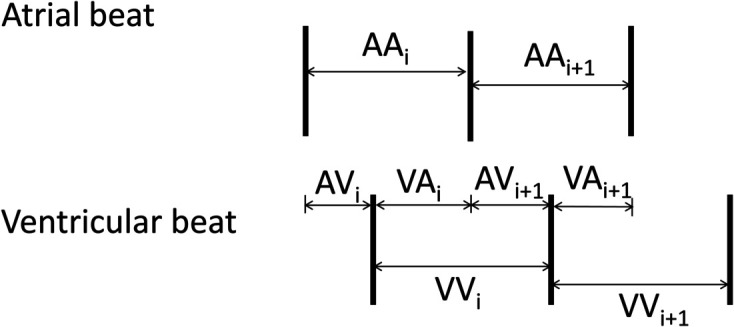
Sequence of atrial and ventricular beats, and corresponding interbeat intervals.

By definition (Fig 4), the V-V (or R-R) interval is described by the difference equation

$$VV_{i+1}=AA_{i}+AV_{i+1}-AV_{i}$$ (16)

Then, the filtering effect of the AV-node is governed by a simple logic: if the recovery time is less than the ventricular refractory time, *θ*, the signal through the AV node is blocked. Otherwise, the next V-V interval is calculated from Eqs 15 and 16. That is, a distribution of R-R intervals during AF, $T_{dist}^{\left(AF\right)}$, can be obtained by iterating through the algorithm

$$\text{if }VA_{i}<\theta \;\;AV_{i+1}=h(VA_{i})\;\text{else}\;\;VA_{i+1}=VA_{i}+AA_{i}\;\;VV_{i+1}=AA_{i}+AV_{i+1}-AV_{i}$$ (17)

In our original model, the change in successive heart periods was defined as the sum of efferent sympathetic, $\Delta T_{s}$, and vagal, $\Delta T_{v}$ contributions. If a realization from $T_{dist}^{\left(AF\right)}$ is denoted as *T*^(*AF*)^, the heart period during a cycle *k* + 1 during AF can be expressed as

$$T_{k+1}=T_{k}+\Delta T_{s,k}+\Delta T_{v,k}+T^{\left(AF\right)}-{\bar{T}}^{\left(AF\right)}$$ (18)

where ${\bar{T}}^{\left(AF\right)}$ is the expected value of $T_{dist}^{\left(AF\right)}$.

### AF-induced changes to the baroreflex

The effect of AF is not limited to changes in the left heart. Baroreflex sensitivity (BRS), a metric of the intrinsic control system’s ability to respond to beat-to-beat changes in arterial pressure [35], is reduced during AF and deteriorates further with the progression from paroxysmal to persistent AF [9,36]. Pharmacological methods to measure BRS employ some variation of the Oxford technique [37] that involves the injection of a vasoconstrictor (typically phenylephrine) to induce a heightened blood pressure and in turn, trigger a parasympathetic-mediated lengthening of the heart period. BRS is then defined as the gradient of the linear relationship between systolic blood pressure and heart period. In essence, a reduction in BRS as defined by the Oxford method, is an indication of the suppression of parasympathetic tone. However, the mode of AF-induced BRS suppression is unclear. It has been suggested that LA dilatation and fibrosis of the atrial muscle may interfere with the activity of cardiopulmonary pressure receptors on the atrial surface. BRS reduction may also be a result of AF-induced endothelial dysfunction which impairs baroreceptor function [9]. It should be noted that although the restoration of a normal sinus rhythm by catheter ablation or cardioversion also increases BRS, [38] observed that post-cardioversion BRS values remained lower than in healthy groups, indicating that AF co-morbidities are additional sources of BRS suppression.

Neuronal activity within each functional unit of our baroreflex model (Fig 2) is described with sigmoidal functions [17]. The model features three afferent pathways that relay pressure information in the form of action potentials from baroreceptors, cardiopulmonary receptors and lung stretch receptors. Parasympathetic response to maintain homeostasis is generated within functionally distinct regions in nucleus tractus solitarius (NTS), nucleus ambiguus (NA) and dorsal motor nucleus of the vagus (DMV), and implemented via vagal efferent pathways that affect heart period and ventricular elastance. We model changes to BRS by introducing a parameter, *k*>0, to the sigmoidal functions that describe baroreceptor and cardiopulmonary receptor activities such that the parasympathetic response of the baroreflex is increasingly muted or boosted at values of *k* > 1 and *k* < 1 respectively

$$f_{out,i}=\frac{f_{min,i}+f_{max,i}e^{\frac{P_{i}-P_{mid,i}}{k\cdot k_{i}}}}{1+e^{\frac{f_{i}-P_{mid,i}}{k\cdot k_{i}}}}\;i=br,\;cpr$$ (19)

where *f*_*out*,*i*_ the spike rates of action potentials from the baroreceptors and cardiopulmonary receptors respectively, *f*_*max*,*i*_ and *f*_*min*,*i*_ are the respective maximum and minimum spike rates, and *P*_*mid*,*i*_ are the pressure values that correspond to the mid-point spike rates.

## Results

### Baseline hemodynamics and baroreflex sensitivity

**Fig 5:**
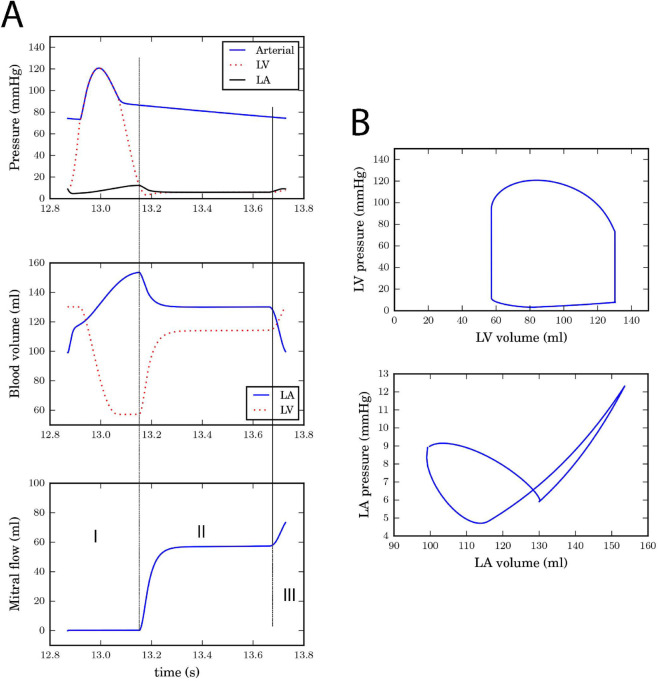
Simulation of baseline hemodynamics under rest conditions. A: Simulation of the baseline left atrial and ventricular pressure, blood volume and transmitral flow during a full heart cycle. Stages I-III depict the reservoir, conduit and contraction roles of the left atrium (LA). B: Corresponding pressure-volume plots of the left ventricle (LV) and left atrium (LA).

**Fig 6:**
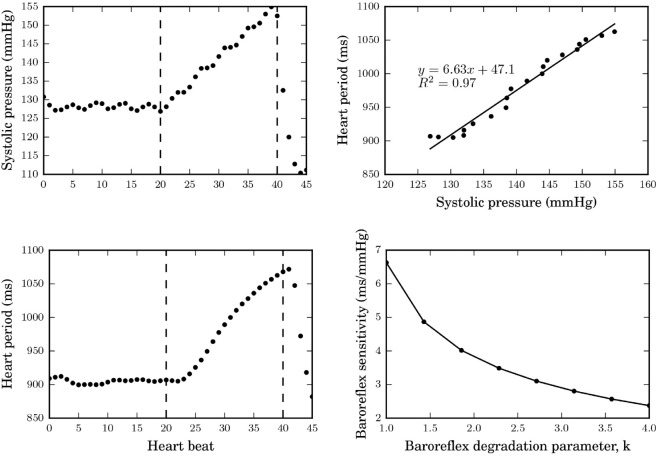
Determination of baroreflex sensitivity (BRS). Top left and bottom left: Simulation of vasoconstrictor-induced rise in systolic pressure and corresponding increase in heart period. Top right: The slope of the linear relationship between the heart period and systolic pressure response determines BRS. Bottom right: The trend in BRS as the degradation parameter, *k* increases.

To solve the parameter tuning problem of Eq 9, the differential equations that describe the improved but still deterministic modified cardio-baroreflex system was solved with each candidate parameter set using the forward-stepping Euler integration method with Δt=0.001s. An optimal solution is provided in Table 1. This set of optimal parameters was used to simulate baseline hemodynamic behavior over a complete heart cycle during rest as shown in Fig 5 with the sequential reservoir (I), conduit (II) and contraction (III) roles of the LA clearly depicted. In order to explore the quantitative relationship between the degradation parameter, *k*, and BRS, we mimicked the effect of injecting a vasoconstrictor into the blood stream by introducing perturbations to the systemic cardiovascular (splanchnic, extra-splanchnic, and active muscle) resistances (Fig 2). Simulations of the rise in systolic blood pressure with the subsequent heart period response, and the monotonic decrease in BRS as *k* increases from its baseline value (*k* = 1) are summarized in Fig 6.

**Table 1:** Optimal pulmonary circulation parameters characterizing reservoir-conduit hysteresis and atrial contraction under rest conditions.

Compliances	Resistances	Left atrium
ml/mmHg	mmHg ⋅ s ⋅ ml^−1^	
*C*_*pa*_ = 0.22	*R*_*pa*_ = 0.086	b = 0.495mmHg
*C*_*pp*_ = 1.865	*R*_*pp*_ = 0.005	0.16 ml^−1^
$C_{pv}=$ 2.98	$R_{pv}=$ 0.0014	*H*_*max*_ = 0.45
*C*_*la*_ = 7.53	*R*_*la*_ = 0.015	$V_{u,la}=31.25$ml

### Effect of AF on select hemodynamic variables

**Fig 7:**
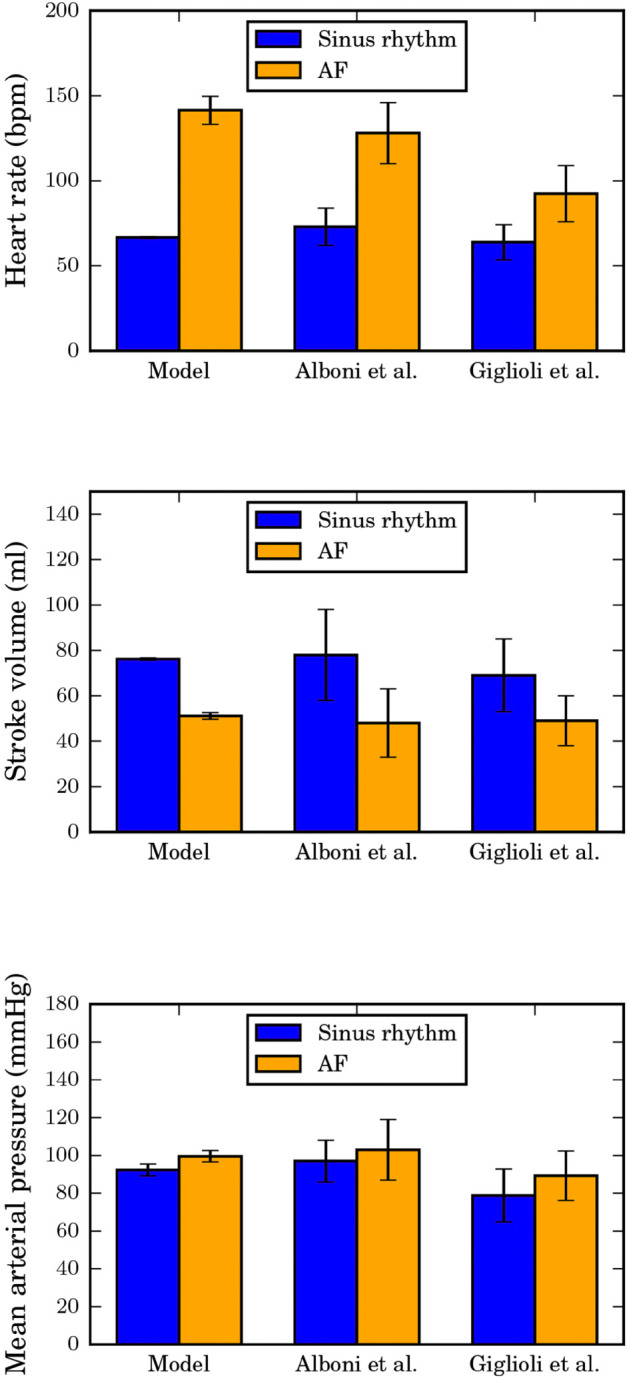
Comparison of model predictions with clinical AF observations. Top: heart rate, middle: stroke volume and bottom: mean arterial pressure). The error bars represent standard deviations.

**Fig 8:**
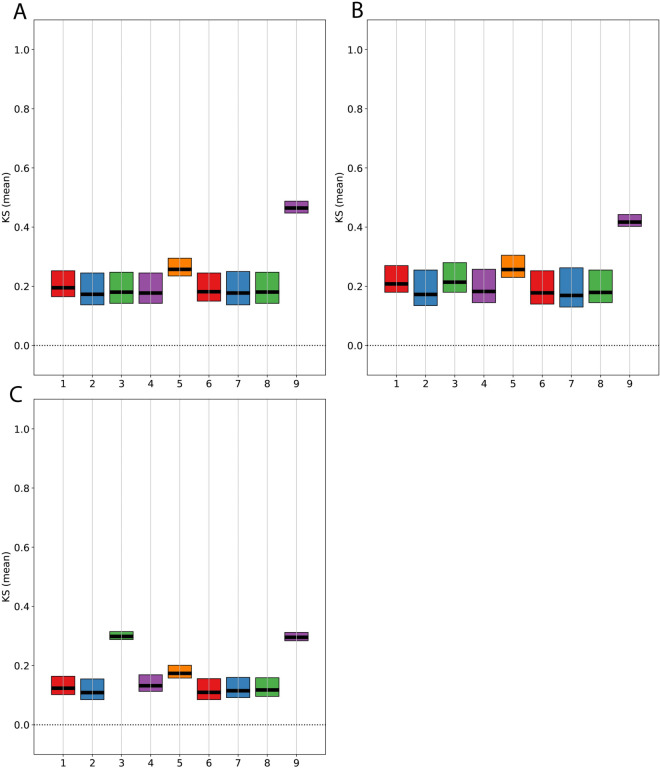
Sensitivities of hemodynamic quantities to ±20% perturbation in model parameters. (A) Heart rate, (B) Stroke volume, and (C) Mean arterial pressure. Parameters 1-9 are defined in Table 2. KS (mean) refers to the mean of the Kolmogorov-Smirnov statistic used to assess the relative influence of input parameters on model outputs in Pianosi and Wagener’s sensitivity analysis tool [41].

**Fig 9:**
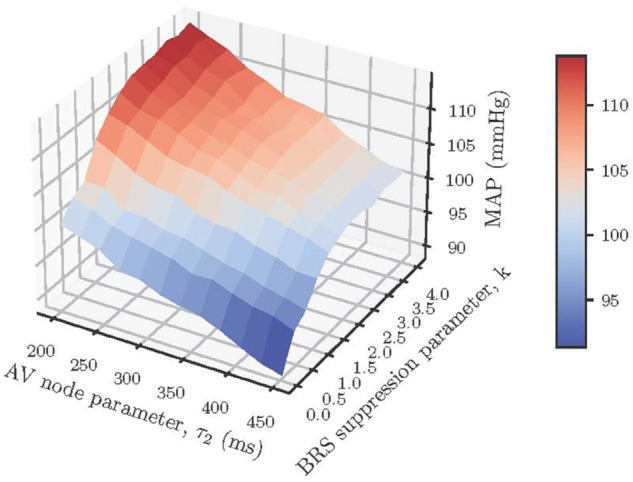
Effect of baroreflex impairment and AV node delay on mean arterial pressure (MAP). MAP response varies from normal to slightly elevated with variation in baroreflex impairment, *k*, and AV node delay, $\tau_{2}$.

In this section, we examine model predictions of changes in key hemodynamic parameters: heart rate, mean arterial pressure and stroke volume during atrial fibrillation. Instances of AF were simulated by inducing a loss of atrial kick and some degree of baroreflex suppression (*k* was set to 4) according to Eqs 14 and 19 respectively. We also introduced randomness to the interatrial and interventricular intervals by implementing the AV filtering algorithm described in Eq 17. In other words, the extent of baroreflex suppression, the distribution of interatrial period, (AA) and AV-node parameters are considered as model inputs. Nominal values of these parameters are provided in Table 2. Our model predictions of hemodynamic changes due to AF are compared with clinical AF observations [39,40] in Fig 7. We also conducted a global sensitivity analysis with a distribution-based method [41] to identify the most influential parameters for each hemodynamic metric. These results are presented in Figs 8 and 9.

**Table 2:** Atrioventricular recovery curve [34] and baroreflex suppression parameters.

Index	Parameter	Description	Nominal value
1	$\bar{AA}$	Mean of interatrial period distribution	150ms
2	$\sigma_{AA}$	Spread of interatrial period distribution	30ms
3	*k*	Baroreflex suppression parameter	4
4	*θ*	AV refractory period	60ms
5	*PR*_*min*_	Minimum AV conduction time	191ms
6	$\alpha_{1}$	Maximum conduction time (fast pathway)	1455ms
7	$\tau_{1}$	Time constant (fast pathway)	11ms
8	$\alpha_{2}$	Maximum conduction time (slow pathway)	161ms
9	$\tau_{2}$	Time constant (slow pathway)	341ms

## Discussion

The simulation of the improved hemodynamic model under sinus rhythm provided in Fig 5 shows that the relevant hemodynamic variables of interest: heart rate (67 beats per minute), stroke volume (85ml, with an atrial kick contribution of 20ml) and mean arterial pressure (93.5mmHg) all lie within clinically-acceptable ranges. Similarly, our model estimate of baseline baroreflex sensitivity (BRS), 6.63 ms/mmHg in Fig 6, is above the 3 ms/mmHg threshold for normal subjects using the phenylephrine method [35]. In addition to cardiovascular disease, age is known to be a significant contributor to reduction in BRS as the gradual loss of arterial elasticity contributes to BRS reduction in older subjects [35]. However, the consideration of the subject’s age as a model input is beyond the scope of this work but an important avenue for further model development.

Model simulations of AF shown in Fig 7 using the baseline parameters provided in Table 2 show tachycardia (145 beats per minute (bpm)) during AF, compared to 67 bpm during sinus rhythm. Results of the global sensitivity analysis in Fig 8 indicate that the time constant, $\tau_{2}$, (parameter 9) of the slow conduction pathway through the AV-node is the main indicator of the AV node’s filtering ability. A known symptom of AF is dizziness (or in more serious cases, syncope) that arises from a reduced stroke volume (SV). Our baseline AF model predicts an average of 27% reduction in stroke volume compared to a normal sinus rhythm. Similar to heart rate, Fig 8 reveals that the dynamics of the slow conduction pathway of the AV-node is the main driver for stroke volume reduction. This can be explained by the proportionality of atrial and ventricular contraction periods to heart rate such that the duration of passive atrial emptying is reduced at elevated heart rates. During heightened physical activity in a normal heart, an increase in left ventricular preload triggers a concomitant increase in the force of atrial contraction to maintain adequate blood flow to the left ventricle. However, full atrial contractions are absent during AF so there is a marked reduction in systemic blood flow at higher heart rates.

While there is general agreement on the effects of AF on heart rate and stroke volume, observations of the effect of AF on mean arterial pressure range from hypotension to hypertension [16]. The wide range of blood pressure outcomes during standalone AF may be explained simply by the competing effects of reduction in stroke volume and suppression of parasympathetic tone. That is, a drop in arterial pressure can be a direct result of a reduction in systemic blood flow. On the other hand, a reduction in BRS increases the relative sympathetic tone which can cause hypertension. This theory is supported by the global sensitivity analysis result in Fig 8 where the baroreflex degradation (parameter 3), along with $\tau_{2}$ which influences heart rate and stroke volume, are identified as the most important predictors of the blood pressure response during AF. We explored this notion further by varying $\tau_{2}$ and *k* to generate the mean arterial pressure (MAP) response surface shown in Fig 9. The plot suggests that a parasympathetic boost, possibly by chemical or electrical stimulation (*k*<1), can counteract the naturally hypertensive effects of an AF-compromised (shortened slow pathway conduction) AV node, resulting in normotensive AF.

The general agreement between our simulation results with clinical data buttresses our assertion that a lumped parameter, statistical distribution approach to modeling chaos in the AV node during AF is sufficient to predict downstream hemodynamic impact. However, the consideration of spatial variation of electrical activity within the heart’s chambers remains crucial in the analysis of AFib initiation, propagation and maintenance. The identification of the AV slow conduction pathway as the primary determinant of hemodynamic impact is noteworthy as radiofrequency ablation of the slow pathway is already considered a viable route for heart rate control in instances of drug refractory AF [42,43]. Indeed, the modification of AV conduction to improve outcomes in cardiovascular disease management is not a novel concept. Atrioventricular delay optimization (AVO) has been determined to improve left ventricular systolic function in patients with biventricular pacemakers for cardiac resynchronization therapy [44–46]. There is also growing interest in the alteration of AV-node conduction in the management of cardiovascular disease via electrical stimulation of the vagus. For example, a low frequency stimulation of the cervical branch of the vagus, which innervates the AV-node, has been demonstrated to halt and even reverse AF in Wistar rats [15]. Similarly, intermittent vagal nerve stimulation (VNS) has been shown to reduce the burden of AF in humans [47]. Since our model already captures the regulatory function of the baroreflex, including the parasympathetic effect of vagal efferents on heart rate, we intend to further explore the vagal-AV node relationship in more detail in future studies. This expanded model will enable in-silico investigations into the design of neural stimulation protocols for AF management using our recently developed CONTROL-CORE simulation platform [48].

## Conclusion

We have presented a new computational model for the prediction of hemodynamic variables following an onset of atrial fibrillation. Crucially, our closed-loop model captures the regulatory function (impaired or otherwise) of the baroreflex and is therefore more realistic than those employed in similar studies. To accurately translate the electrophysiological changes caused by AF to measurable hemodynamic effects, we made modifications to the base model of [17]. First, we refined left atrial operation to account for atrial contraction and hysteresis between the reservoir and conduit stages. Then, an onset of fibrillation was described by the removal of atrial kick and partial suppression of the parasympathetic tone through the introduction of a new parameter that quantifies the degree of impairment to the baroreflex. In addition, the pacemaking function of the atrioventricular node during AF was described using a stochastic difference equation. Our model predictions of heart rate, stroke volume and mean arterial pressure during AF are in agreement with published clinical observations and provide insight into the wide range of blood pressure outcomes during AF. We also identified the slow conduction pathway of the AV-node and the degree of baroreflex impairment as the key drivers of these hemodynamic quantities.

This model sets the stage for further studies into the effect of age and disease progression (from paroxysmal to chronic AF) on BRS, and the modification of AV-node conductive properties and the direct boost of baroreflex sensitivity via vagal nerve stimulation. Our ultimate goal is the design of an exogenous control loop that regulates the optimal dosage of electrical stimulation based on bio-feedback from the patient as it will undoubtedly be a major step towards personalized AF management. This will require an expansion of our model to the cellular level to include protein biomarkers associated with the incidence of AF.

## Supporting information

S1 File Mathematical description and parameter values of the cardio-baroreflex model. The set of differential algebraic equations that describe our human cardio-baroreflex model under rest conditions and during AF.
(PDF)

## References

[1] Bassand J-P, Virdone S, Goldhaber SZ, Camm AJ, Fitzmaurice DA, Fox KAA, et al. Early risks of death, stroke/systemic embolism, and major bleeding in patients with newly diagnosed atrial fibrillation. Circulation. 2019;139(6):787–98. doi: 10.1161/CIRCULATIONAHA.118.035012 30586740

[2] Morillo CA, Banerjee A, Perel P, Wood D, Jouven X. Atrial fibrillation: the current epidemic. J Geriatr Cardiol. 2017;14(3):195–203. doi: 10.11909/j.issn.1671-5411.2017.03.011 28592963 PMC5460066

[3] Darby AE. Recurrent atrial fibrillation after catheter ablation: considerations for repeat ablation and strategies to optimize success. J Atr Fibrillation. 2016;9(1):1427. doi: 10.4022/jafib.1427 27909521 PMC5089515

[4] Van Gelder IC, Crijns HJ. Cardioversion of atrial fibrillation and subsequent maintenance of sinus rhythm. Pacing Clin Electrophysiol. 1997;20(10 Pt 2):2675–83. doi: 10.1111/j.1540-8159.1997.tb06116.x 9358514

[5] Kondo H, Shinohara T, Fukui A, Miyoshi M, Ishii Y, Otsubo T, et al. Possible role of baroreflex sensitivity in patients with paroxysmal atrial fibrillation. JACC Clin Electrophysiol. 2019;5(4):523–5. doi: 10.1016/j.jacep.2019.01.009 31000109

[6] Xi Y, Cheng J. Dysfunction of the autonomic nervous system in atrial fibrillation. J Thorac Dis. 2015;7(2):193–8. doi: 10.3978/j.issn.2072-1439.2015.01.12 25713736 PMC4321068

[7] Lombardi F, Tarricone D, Tundo F, Colombo F, Belletti S, Fiorentini C. Autonomic nervous system and paroxysmal atrial fibrillation: a study based on the analysis of RR interval changes before, during and after paroxysmal atrial fibrillation. Eur Heart J. 2004;25(14):1242–8. doi: 10.1016/j.ehj.2004.05.016 15246643

[8] Czick ME, Shapter CL, Silverman DI. Atrial fibrillation: the science behind its defiance. Aging Dis. 2016;7(5):635–56. doi: 10.14336/AD.2016.0211 27699086 PMC5036958

[9] Miyoshi M, Kondo H, Ishii Y, Shinohara T, Yonezu K, Harada T, et al. Baroreflex sensitivity in patients with atrial fibrillation. J Am Heart Assoc. 2020;9(24):e018019. doi: 10.1161/JAHA.120.018019 33263265 PMC7955376

[10] Roy A, Varela M, Aslanidi O. Image-based computational evaluation of the effects of atrial wall thickness and fibrosis on re-entrant drivers for atrial fibrillation. Frontiers in Physiology. 2018;9:388346. 10.3389/fphys.2018.01352 PMC6187302 30349483

[11] Roy A, Varela M, Chubb H, MacLeod R, Hancox JC, Schaeffter T, et al. Identifying locations of re-entrant drivers from patient-specific distribution of fibrosis in the left atrium. PLoS Comput Biol. 2020;16(9):e1008086. doi: 10.1371/journal.pcbi.1008086 32966275 PMC7535127

[12] Deng D, Murphy MJ, Hakim JB, Franceschi WH, Zahid S, Pashakhanloo F, et al. Sensitivity of reentrant driver localization to electrophysiological parameter variability in image-based computational models of persistent atrial fibrillation sustained by a fibrotic substrate. Chaos. 2017;27(9):093932. doi: 10.1063/1.5003340 28964164 PMC5605332

[13] McDowell KS, Zahid S, Vadakkumpadan F, Blauer J, MacLeod RS, Trayanova NA. Virtual electrophysiological study of atrial fibrillation in fibrotic remodeling. PLoS One. 2015;10(2):e0117110. doi: 10.1371/journal.pone.0117110 25692857 PMC4333565

[14] Heijman J, Sutanto H, Crijns HJGM, Nattel S, Trayanova NA. Computational models of atrial fibrillation: achievements, challenges, and perspectives for improving clinical care. Cardiovasc Res. 2021;117(7):1682–99. doi: 10.1093/cvr/cvab138 33890620 PMC8208751

[15] Sheng X, Scherlag BJ, Yu L, Li S, Ali R, Zhang Y, et al. Prevention and reversal of atrial fibrillation inducibility and autonomic remodeling by low-level vagosympathetic nerve stimulation. J Am Coll Cardiol. 2011;57(5):563–71. doi: 10.1016/j.jacc.2010.09.034 21272747

[16] Scarsoglio S, Guala A, Camporeale C, Ridolfi L. Impact of atrial fibrillation on the cardiovascular system through a lumped-parameter approach. Med Biol Eng Comput. 2014;52(11):905–20. doi: 10.1007/s11517-014-1192-4 25192922

[17] Park JH, Gorky J, Ogunnaike B, Vadigepalli R, Schwaber JS. Investigating the effects of brainstem neuronal adaptation on cardiovascular homeostasis. Front Neurosci. 2020;14:470. doi: 10.3389/fnins.2020.00470 32508573 PMC7251082

[18] Matsuda Y, Toma Y, Ogawa H, Matsuzaki M, Katayama K, Fujii T, et al. Importance of left atrial function in patients with myocardial infarction. Circulation. 1983;67(3):566–71. doi: 10.1161/01.cir.67.3.566 6821898

[19] Pagel PS, Kehl F, Gare M, Hettrick DA, Kersten JR, Warltier DC. Mechanical function of the left atrium: new insights based on analysis of pressure-volume relations and Doppler echocardiography. Anesthesiology. 2003;98(4):975–94. doi: 10.1097/00000542-200304000-00027 12657862

[20] Pironet A, Dauby PC, Paeme S, Kosta S, Chase JG, Desaive T. Simulation of left atrial function using a multi-scale model of the cardiovascular system. PLoS One. 2013;8(6):e65146. doi: 10.1371/journal.pone.0065146 23755183 PMC3670859

[21] Noreen S, Ben-Tal A, Elstad M, Sweatman WL, Ramchandra R, Paton J. Mathematical modelling of atrial and ventricular pressure-volume dynamics and their change with heart rate. Math Biosci. 2022;344:108766. doi: 10.1016/j.mbs.2021.108766 34919936

[22] Arakawa M, Tanaka T, Hirakawa S. Pressure-volume relation of the left atrium in man. Cardiac mechanics and function in the normal and diseased heart. Japan: Springer; 1989. p. 147–54. 10.1007/978-4-431-67957-8_15

[23] Karanasos A, Tyrovolas K, Tsiachris D, Efremidis M, Kordalis A, Karmpalioti M, et al. Left atrial function post radiofrequency and cryoballoon ablation assessed by volume-pressure loops. Front Cardiovasc Med. 2022;9:830055. doi: 10.3389/fcvm.2022.830055 35355975 PMC8959489

[24] Ursino M. Interaction between carotid baroregulation and the pulsating heart: a mathematical model. Am J Physiol. 1998;275(5):H1733-47. doi: 10.1152/ajpheart.1998.275.5.H1733 9815081

[25] Carruthers SG, McCall B, Cordell BA, Wu R. Relationships between heart rate and PR interval during physiological and pharmacological interventions. Br J Clin Pharmacol. 1987;23(3):259–65. doi: 10.1111/j.1365-2125.1987.tb03043.x 2882775 PMC1386222

[26] Joe S, Kuo FY. Remark on algorithm 659: Implementing Sobol’s quasirandom sequence generator. ACM Transactions on Mathematical Software. 2003;29(1):49–57.

[27] Sanfilippo AJ, Abascal VM, Sheehan M, Oertel LB, Harrigan P, Hughes RA, et al. Atrial enlargement as a consequence of atrial fibrillation. A prospective echocardiographic study. Circulation. 1990;82(3):792–7. doi: 10.1161/01.cir.82.3.792 2144217

[28] Tateno K, Glass L. Automatic detection of atrial fibrillation using the coefficient of variation and density histograms of RR and deltaRR intervals. Med Biol Eng Comput. 2001;39(6):664–71. doi: 10.1007/BF02345439 11804173

[29] Bootsma BK, Hoelsen AJ, Strackee J, Meijler FL. Analysis of R-R intervals in patients with atrial fibrillation at rest and during exercise. Circulation. 1970;41(5):783–94. doi: 10.1161/01.cir.41.5.783 5429488

[30] Hayano J, Yamasaki F, Sakata S, Okada A, Mukai S, Fujinami T. Spectral characteristics of ventricular response to atrial fibrillation. Am J Physiol. 1997;273(6):H2811-6. doi: 10.1152/ajpheart.1997.273.6.H2811 9435618

[31] Hong-Wei L, Ying S, Min L, Pi-Ding L, Zheng Z. A probability density function method for detecting atrial fibrillation using R-R intervals. Med Eng Phys. 2009;31(1):116–23. doi: 10.1016/j.medengphy.2008.04.013 18554974

[32] Lian J, Wang L, Muessig D. A simple method to detect atrial fibrillation using RR intervals. Am J Cardiol. 2011;107(10):1494–7. doi: 10.1016/j.amjcard.2011.01.028 21420064

[33] Vaiciulyte OR, Lekas R, Civinskiene G, Lekas V, Andriuskevicius J, Bernatoniene J. Deciding on modification of the atrioventricular node using the RR interval histogram. E-journal of Cardiology Practice Sophia Antipolis: European Society of Cardiology 2013;11:16.

[34] Zeng W, Glass L. Statistical properties of heartbeat intervals during atrial fibrillation. Phys Rev E Stat Phys Plasmas Fluids Relat Interdiscip Topics. 1996;54(2):1779–84. doi: 10.1103/physreve.54.1779 9965257

[35] La Rovere MT, Pinna GD, Raczak G. Baroreflex sensitivity: measurement and clinical implications. Ann Noninvasive Electrocardiol. 2008;13(2):191–207. doi: 10.1111/j.1542-474X.2008.00219.x 18426445 PMC6931942

[36] Wasmund SL, Li J-M, Page RL, Joglar JA, Kowal RC, Smith ML, et al. Effect of atrial fibrillation and an irregular ventricular response on sympathetic nerve activity in human subjects. Circulation. 2003;107(15):2011–5. doi: 10.1161/01.CIR.0000064900.76674.CC 12681998

[37] Smyth HS, Sleight P, Pickering GW. Reflex regulation of arterial pressure during sleep in man. A quantitative method of assessing baroreflex sensitivity. Circ Res. 1969;24(1):109–21. doi: 10.1161/01.res.24.1.109 4303309

[38] Field ME, Wasmund SL, Page RL, Hamdan MH. Restoring sinus rhythm improves baroreflex function in patients with persistent atrial fibrillation. J Am Heart Assoc. 2016;5(2):e002997. doi: 10.1161/JAHA.115.002997 26908410 PMC4802450

[39] Alboni P, Scarfò S, Fucà G, Paparella N, Yannacopulu P. Hemodynamics of idiopathic paroxysmal atrial fibrillation. Pacing Clin Electrophysiol. 1995;18(5 Pt 1):980–5. doi: 10.1111/j.1540-8159.1995.tb04738.x 7659571

[40] Giglioli C, Nesti M, Cecchi E, Landi D, Chiostri M, Gensini GF, et al. Hemodynamic effects in patients with atrial fibrillation submitted to electrical cardioversion. Int J Cardiol. 2013;168(4):4447–50. doi: 10.1016/j.ijcard.2013.06.150 23890895

[41] Pianosi F, Wagener T. A simple and efficient method for global sensitivity analysis based on cumulative distribution functions. Environmental Modelling & Software. 2015;67:1–11.

[42] Della Bella P, Carbucicchio C, Tondo C, Riva S. Modulation of atrioventricular conduction by ablation of the “slow” atrioventricular node pathway in patients with drug-refractory atrial fibrillation or flutter. J Am Coll Cardiol. 1995;25(1):39–46. doi: 10.1016/0735-1097(94)00315-h 7798523

[43] Canby RC, Román CA, Kessler DJ, Horton RP, Page RL. Selective radiofrequency ablation of the “slow” atrioventricular nodal pathway for control of the ventricular response to atrial fibrillation. Am J Cardiol. 1996;77(15):1358–61. doi: 10.1016/s0002-9149(96)00206-8 8677880

[44] Whinnett ZI, Davies JER, Willson K, Manisty CH, Chow AW, Foale RA, et al. Haemodynamic effects of changes in atrioventricular and interventricular delay in cardiac resynchronisation therapy show a consistent pattern: analysis of shape, magnitude and relative importance of atrioventricular and interventricular delay. Heart. 2006;92(11):1628–34. doi: 10.1136/hrt.2005.080721 16709698 PMC1861257

[45] Sawhney NS, Waggoner AD, Garhwal S, Chawla MK, Osborn J, Faddis MN. Randomized prospective trial of atrioventricular delay programming for cardiac resynchronization therapy. Heart Rhythm. 2004;1(5):562–7. doi: 10.1016/j.hrthm.2004.07.006 15851220

[46] Auricchio A, Stellbrink C, Block M, Sack S, Vogt J, Bakker P, et al. Effect of pacing chamber and atrioventricular delay on acute systolic function of paced patients with congestive heart failure. The Pacing Therapies for Congestive Heart Failure Study Group. The Guidant Congestive Heart Failure Research Group. Circulation. 1999;99(23):2993–3001. doi: 10.1161/01.cir.99.23.2993 10368116

[47] Stavrakis S, Stoner JA, Humphrey MB, Morris L, Filiberti A, Reynolds JC, et al. TREAT AF (Transcutaneous Electrical Vagus Nerve Stimulation to Suppress Atrial Fibrillation): a randomized clinical trial. JACC Clin Electrophysiol. 2020;6(3):282–91. doi: 10.1016/j.jacep.2019.11.008 32192678 PMC7100921

[48] Kathiravelu P, Arnold M, Fleischer J, Yao Y, Awasthi S, Goel AK, et al. CONTROL-CORE: a framework for simulation and design of closed-loop peripheral neuromodulation control systems. IEEE Access. 2022;10:36268–85. doi: 10.1109/access.2022.3161471 36199437 PMC9531851

