# Supplementary material for: Short term hemodynamic effects of atrial fibrillation in a closed-loop human cardiac-baroreflex system
Source: PLoS One. 2025 Oct 29;20(10):e0334086. doi: 10.1371/journal.pone.0334086 (PMC12571259; doi:10.1371/journal.pone.0334086)
Supplement: S1 File — The set of differential algebraic equations that describe our human cardio-baroreflex model under rest conditions and during AF. (PDF) [file pone.0334086.s001.pdf]

Supplementary material for 'Short term hemodynamic effects of atrial fibrillation in a closed-loop human cardiac-baroreflex system'

Oluwasanmi Adeodu[1], Michelle Gee[2], Babak Mahmoudi[3], Rajanikanth Vadigepalli[4], Mayuresh Kothare[1*]

1 Department of Chemical and Biomolecular Engineering, Lehigh University, Bethlehem, United States
2 Department of Chemical and Biomolecular Engineering, University of Delaware, Newark, United States
3 Department of Biomedical Engineering, Emory University, United States
4 Department of Pathology, Anatomy and Cell Biology, Thomas Jefferson University, United States

* mayuresh.kothare@lehigh.edu

Mathematical model of the cardiovascular system

In this supplementary text, we provide the equations and parameters describing the evolution of baroreflex-controlled outputs and cardiovascular pressures (or volumes) in the compartments of the cardio-baroreflex system (Fig 1) from Park et al. [1]. We also highlight atrial fibrillation (AF)-specific modifications made in the current paper. The equations constitute the nonlinear, algebraic ordinary differential system that describe the cardio-baroreflex model and is represented in discrete form by the dynamical model f in Equation 7 of the main text. In other words, equations (7 - 43) in this supplementary text, along with the stated modifications, correspond to

$$x_{i+1} = f(x_i, p) \tag{1}$$

where i is the heart cycle index; the vector of states, x, comprises of the pressures (or volumes) of the various compartments in the cardiovascular system, splanchnic and extrasplanchnic resistances, sympathetic and vagal contributions to heart rate and ventricular contractility. The vector p comprises of resistance and compliance terms within the pulmonary circulation system. It also includes the newly introduced quantities, H_{max} and Ela, that describe a realistic left atrial function.

$$y_i = g(x_i, p) \tag{2}$$

The function, g, in Equation 2 (Equation 8 in the main text) is the vector of functions that yield y, the hemodynamic variables of interest: (heart rate, mean arterial pressure, stroke volume and left atrial end systolic volume (LAESV)). Heart rate is the reciprocal of the heart period, i.e

$$H(\text{beats per minute}) = \frac{60}{T} \tag{3}$$

Mean arterial pressure (MAP) is calculated from the diastolic (D) and systolic (S) pressures as

$$MAP = \frac{2D + S}{3} \tag{4}$$

Stroke volume (SV) is the difference between the left ventricular end-diastolic (ED) and systolic (ES) volumes

$$SV = ED - ES \tag{5}$$

LAESV is the maximum volume of the left atrium during a heart cycle :

$$LAESV = max(\text{left atrial volume}) \tag{6}$$

Therefore, Equations (3 - 6) constitute the function g.

The heart as a pump

The equations in this section are obtained from mass and force (for large arteries) balances across each compartment. P_i is intravascular pressure in the ith compartment, $V_{u,i}$ is the corresponding unstressed volume; C_i, L_i, and R_i are the compliances, inertances, and hydraulic resistances, respectively. $F_{i,r}$ and $F_{i,l}$ are volumetric flow rates into the left and right ventricles respectively. Similarly, $F_{o,r}$ and $F_{o,l}$ are the cardiac outputs from the left and right ventricles.

Balance across the left atrium:

$$\dot{P}_{la} = \frac{1}{C_{la}}\left(\frac{P_{pv} - P_{la}}{R_{pv}} - F_{il}\right) \tag{7}$$

$$F_{i,l} = \begin{cases} 0 & \text{if } P_{la} \leq P_{lv}, \\ \frac{P_{la} - P_{lv}}{R_{la}} & \text{if } P_{la} > P_{lv}. \end{cases} \tag{8}$$

Modification 1: In the current paper, Equation (7) is modified to capture atrial contraction, and the distinct pressure-volume pathways of the left atrium (LA) during passive filling and emptying.

Balance across the left ventricle:

$$\dot{V}_{lv} = F_{i,l} - F_{o,l} \tag{9}$$

$$F_{o,l} = \begin{cases} 0 & P_{max,lv} \leq P_{sa}, \\ \frac{P_{max,lv} - P_{sa}}{R_{lv}} & P_{max,lv} > P_{sa} \end{cases} \tag{10}$$

. The left ventricular isometric pressure, $P_{max,lv}$ is defined as

$$P_{max,lv}(t) = \varphi(t) \cdot E_{max,lv}(t) \cdot (V_{lv} - V_{u,lv}) + [1 - \varphi(t)] \cdot \left[P_{0,lv} \cdot \left(e^{k_{E,lv} \cdot V_{lv} - 1}\right)\right] \tag{11}$$

where $\varphi(t)$ is the activation function that describes the periodic contraction of the ventricles such that $\varphi = 1$ at systole and $\varphi = 0$ during diastole. $E_{max,lv}$ is the instantaneous maximum elastance of the left ventricle, and $P_{0,lv}$, $k_{E,lv}$ are constants (provided in Table 1) that describe the exponential pressure-volume relationship at diastole.

$$\varphi(t) = \begin{cases} \sin^2\left[\frac{\pi \cdot T(t) \cdot u}{T_{sys}(t)}\right], & 0 \leq u \leq \frac{T_{sys}}{T}, \\ 0, & u > \frac{T_{sys}}{T} \end{cases} \tag{12}$$

where T_{sys} is the duration of systole and is defined to decrease linearly with heart rate.

$$T_{sys} = T_{sys,0} - k_{sys} \cdot \frac{1}{T} \tag{13}$$

$T_{sys,0}$ and k_{sys} are constants. A set of equations analogous to (9)-(12) describes the right ventricle. The introduction of u defines a new differential equation

$$\dot{u} = \frac{1}{T} \tag{14}$$

where $u = 0$ at systole and $u = 1$ corresponds to diastole. That is, u resets to 0 once it reaches the value of 1.

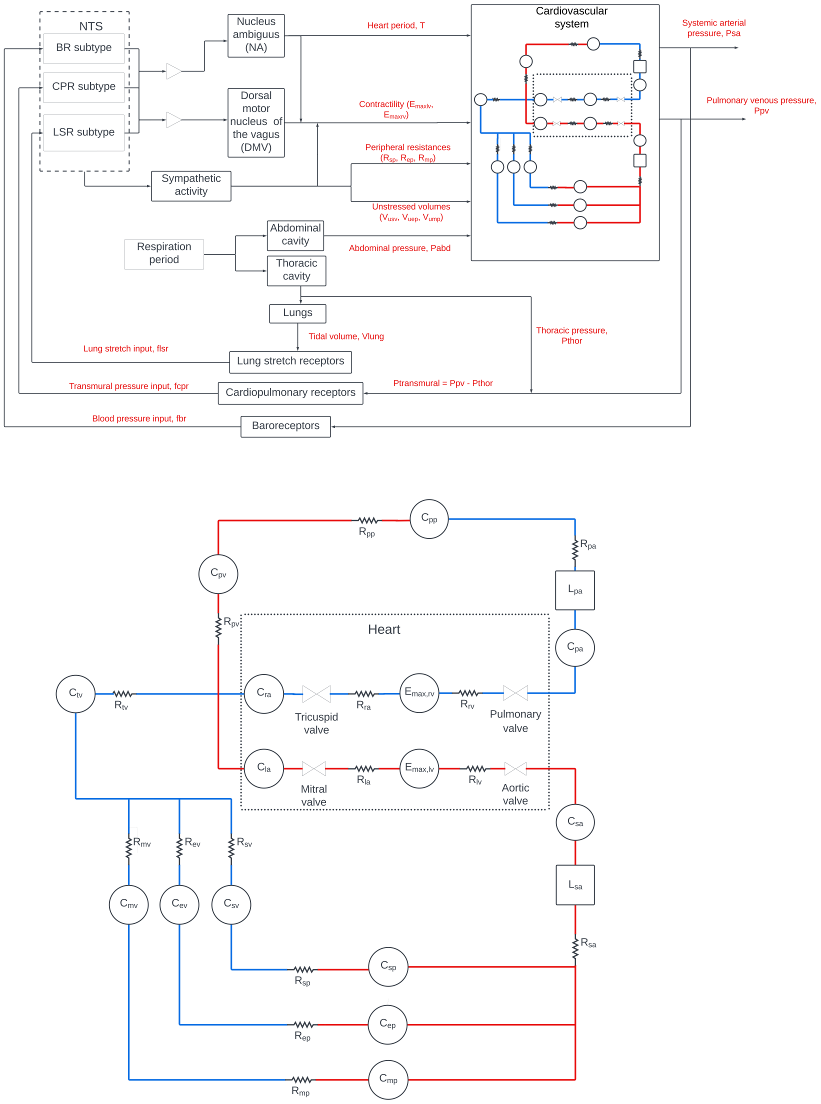

Fig 1. Left: Systemic and pulmonary circulation of the human cardiovascular system. Right: A hydraulic analog of the Park et al. [1] model. Red, oxygenated blood; blue, de-oxygenated blood; P, pressures; R, hydraulic resistances; C, compliances; L, inertances; sa, systemic arteries; sp, splanchnic peripheral circulation, sv, splanchnic venous circulation; ep, extrasplanchnic peripheral circulation, ev, extrasplanchnic venous circulation; mp, muscular peripheral circulation, mv muscular venous circulation; ra, right atrium; rv, right ventricle; pa, pulmonary arteries; pp, pulmonary peripheral circulation; pv, pulmonary veins; la, left atrium; lv, left ventricle

Table 1. Parameters of the left and right heart

Left ventricle	Right ventricle
$C_{la} = 19.23$ ml/mmHg	$C_{ra} = 19.23$ml/mmHg
$V_{u,la} = 25$ml	$V_{u,ra} = 25$ml
$R_{la} = 2.5 \cdot 10^{-3}$ mmHg $\cdot$s$\cdot$ml^{-1}	$R_{ra} = 2.5 \cdot 10^{-3}$ mmHg $\cdot$ s$\cdot$ ml^{-1}
$P_{0,lv} = 1.5$ mmHg	$P_{0,rv} = 1.5$ mmHg
$K_{E,lv} = 0.014$ ml^{-1}	$K_{E,rv} = 0.011$ ml $^{-1}$
$V_{u,lv} = 16.77$ ml	$V_{u,rv} = 40.8$ ml
$k_{R,lv} = 3.75 \cdot 10^{-4}$ s/ml	$k_{R,rv} = 1.4 \cdot 10^{-3}$ s/ml

Other cardiovascular compartments

The hemodynamic parameters of the other passive compartments are provided in Table 2.

Table 2. Parameters of the vascular system

Compartment	Compliance ml/mmHg	Unstressed volume, ml	Resistance mmHg$\cdot$s $\cdot$ml^{-1}	Inertance mmHg$\cdot$ml$\cdot$s^{-2}
sa	0.28	0	0.06	$0.22 \cdot 10^{-3}$
sp	2.05	274.4	3.307	
ep	1.36	274.1	1.725	
mp	0.31	62.5	4.13	
sv	43.11	986.48	0.038	
ev	28.4	484	0.0197	
mv	6.6	93.1	0.0848	
tv	33	0	0.0054	
pa	0.67	0	0.023	$0.18 \cdot 10^{-3}$
pp	5.80	123	0.0894	
pv	25.37	120	0.0056	

Conservation of mass at pulmonary arteries:

$$\dot{P}_{pa} = \frac{1}{C_{pa}} \left(F_{o,r} - F_{pa}\right). \tag{15}$$

Balance of forces at pulmonary arteries:

$$\dot{F}_{pa} = \frac{1}{L_{pa}} \left(P_{pa} - P_{pp} - R_{pa} \cdot F_{pa}\right). \tag{16}$$

Conservation of mass at pulmonary peripheral circulation:

$$\dot{P}_{pp} = \frac{1}{C_{pp}} \left(F_{pa} - \frac{P_{pp} - P_{pv}}{R_{pp}}\right). \tag{17}$$

Conservation of mass at pulmonary veins:

$$\dot{P}_{pv} = \frac{1}{C_{pv}} \left(\frac{P_{pp} - P_{pv}}{R_{pp}} - \frac{P_{pv} - P_{la}}{R_{pv}}\right). \tag{18}$$

Conservation of mass at systemic arteries:

$$\dot{P}_{sa} = \frac{1}{C_{sa}} \left(F_{o,l} - F_{sa}\right). \tag{19}$$

Balance of forces at systemic arteries:

$$\dot{F}_{sa} = \frac{1}{L_{sa}} \left(P_{sa} - P_{sp} - R_{sa} \cdot F_{sa} \right). \tag{20}$$

Conservation of mass at peripheral systemic circulation:

$$\dot{P}_{sp} = \frac{1}{C_{sp} + C_{ep} + C_{mp}} \left(F_{sa} - \frac{P_{sp} - P_{sv}}{R_{sp}} - \frac{P_{sp} - P_{ev}}{R_{ep}} - \frac{P_{sp} - P_{mv}}{R_{mp}} \right). \tag{21}$$

Conservation of mass at extrasplanchnic venous circulation:

$$\dot{P}_{ev} = \frac{1}{C_{ev}} \left(\frac{P_{sp} - P_{ev}}{R_{ep}} - \frac{P_{ev} - P_{tv}}{R_{ev}} - \dot{V}_{u,ev} \right). \tag{22}$$

Conservation of mass at the thoracic venous circulation:

$$\dot{P}_{tv} = \frac{1}{C_{tv}} \left(V_{om} + \frac{P_{ev} - P_{tv}}{R_{ev}} + \frac{P_{sv} - P_{tv}}{R_{sv}} - \frac{P_{tv} - P_{ra}}{R_{tv}} \right) \tag{23}$$

Conservation of mass at the right atrium:

$$\dot{P}_{ra} = \frac{1}{C_{ra}} \left(\frac{P_{tv} - P_{ra}}{R_{tv}} - Q_{i,r} \right). \tag{24}$$

Blood volume in the splanchnic venous circulation is obtained as the difference between total blood volume , V_t and the total volume in other chambers:

$$P_{sv} = \frac{1}{C_{sv}} \left(V_t - V_{lv} - V_{rv} \sum_i C_i P_i - V_{u,i} \right) \tag{25}$$

where i refers to the subscripts of the passive compartments.

Mathematical model of the baroreflex

Afferent nerves projecting from high pressure receptors (baroreceptors) in the carotid sinus, low pressure receptors in the atria (cardiopulmonary receptors) and lung stretch receptors relay pressure and tidal volume information in the form of action potentials to distinct control centers within the brainstem. Hemodynamic regulation is then effected via efferent sympathetic and parasympathetic pathways as illustrated in Fig 1.

Afferent pathways

Pressure information from the baroreceptors and cardiopulmonary receptors are converted to spike rates in the respective afferent fibers via sigmoidal functions.

Baroreceptor afferent pathway

$$\dot{\tilde{P}} = \frac{1}{\tau_p} \left(P_{sa} + \tau_z \dot{P}_{sa} - \tilde{P} \right) \tag{26}$$

$$f_{br} = \frac{f_{min} + f_{max} e^{\frac{\tilde{P} - P_n}{k_a}}}{1 + e^{\frac{\tilde{P} - P_n}{c \cdot k_a}}}. \tag{27}$$

where f_{br} is the firing rate of the afferent signal and $\tilde{P}$ is the intrasinus pressure. The parameters P_n, f_{max}, f_{min} and k_a describe the shape of the sigmoidal function and are provided in Table 3.

Cardiopulmonary afferent pathway

Similarly, f_{cpr} is the firing rate in the cardiopulmonary afferent fibers and is dependent on the transmural pressure in the pulmonary vein:

$$\dot{P}_l = \frac{1}{\tau_{cp}} \left(P_{pv} - P_{thor} - P_l\right) \tag{28}$$

$$f_{cpr} = \frac{f_{max,l}}{1 + e^{\frac{Ptn - Pl}{k_l}}} \tag{29}$$

where P_{thor} is the thoracic pressure. The time constant, τ_{cp}, and the parameters of the sigmoid in (29) are provided in Table 3. **Modification 2: k_a and k_l in Equations 27 and 29 are modified with a multiplicative factor, $k > 0$, with $k > 1$ describing AF-induced suppression of the parasympathetic response of the baroreflex as a result of baroreceptor malfunction, and $k < 1$ describing a boost in parasympathetic tone.**

Lung stretch afferent pathway

The firing rate in the lung stretch fibers is described with a simple ordinary differential equation:

$$\dot{f}_{lsr} = \frac{1}{\tau_{lung}} \left(G_{al} \cdot v_{lung} - f_{lsr}\right) \tag{30}$$

where v_{lung} is the instantaneous tidal volume. Other parameters of Equation 30 are provided in Table 3.

Table 3. Parameters of the afferent pathways

Baroreceptors	Cardiopulmonary receptors	Lung stretch receptors
$\tau_p = 2.067$s	$\tau_{cp} = 2$ s	$\tau_{lung} = 2$ s
$\tau_z = 6.37$s	$P_{tn} = 10.8$ mmHg	$G_{al} = 12$
$P_n = 92$ mmHg	$f_{max,l} = 20$ s^{-1}	
$f_{min} = 2.52$ s^{-1}	$k_l = 11.758$ mmHg	
$f_{max} = 47.78$ s^{-1}		
$k_a = 11.758$ mmHg		

Efferent sympathetic activity

A linear combination of the three afferent signals serve as input to a negative monotonic function that yields the corresponding spike rates in the sympathetic efferent fiber.

$$\begin{bmatrix} f_{as,h} \\ f_{as,r} \\ f_{as,v} \end{bmatrix} = \begin{bmatrix} 1 & 2 & -1.541 \\ 1 & 2.5 & 0.33 \\ 1 & 0 & 0 \end{bmatrix} \begin{bmatrix} f_{br} \\ f_{cpr} \\ f_{lsr} \end{bmatrix} \tag{31}$$

$$f_{es,i} = f_{es,\infty} + \left(f_{es,0} - fes_{\infty} \cdot e^{-k_{es} \cdot f_{cs}}\right) \tag{32}$$

where $f_{es,i}$ $(i = h,\ r,\ v)$ represents the spike rates of the signals to the heart, splanchnic resistances and unstressed volumes respectively. The response to the sympathetic

stimulation includes a pure latency, a monotonic logarithmic static function, and a low-pass first-order dynamics

$$\sigma_\theta = \begin{cases} \ln\left[G_\theta f_{es}(t - D_\theta) - f_{es,min} + 1\right], & \text{if } f_{es} \geq f_{es,min}, \\ 0, & \text{otherwise.} \end{cases}$$

.

$$\frac{d\Delta\theta(t)}{dt} = \frac{1}{\tau_\theta} + \sigma_\theta(t) \quad (33)$$

$$\theta(t) = \Delta\theta(t) + \theta_0 \quad (34)$$

where θ denotes the generic controlled parameter (resistances, unstressed volumes, maximum cardiac elastances and heart period), G_θ is a constant gain factor, θ_0 is the output of the static characteristic, τ_θ and D_θ are the time constants and pure latency, respectively, of the mechanism, $f_{es,min}$ is the minimum sympathetic stimulation and $\Delta\theta$ is the parameter change caused by sympathetic stimulation. Values for these parameters are listed in Table 4.

Table 4. Parameters of the effectors in the sympathetic and vagal pathways

θ	Gain	Time constant (s)	Time delay (s)	θ_0
T_s	-0.13	2	2	0.58
$E_{max,lv,s}$	-0.13	2	2	1.283
$E_{max,rv,s}$	-0.22	2	2	0.757
R_{sp}	0.695	6	2	2.49
R_{ep}	0.653	6	2	0.96
R_{mp}	2.81	6	2	4.13
$V_{u,sv}$	-265.4	20	2	1435.4
$V_{u,ev}$	-107.5	20	2	1247
$V_{u,mv}$	-25	6	2	290
T_v	0.09	1.5	0.2	
$E_{max,lv,v}$	0.205	2	0.2	
$E_{max,rv,v}$	0.347	2	0.2	

Efferent parasympathetic (vagal) activity

The control of heart period, T, and maximum ventricular elastances ($E_{max,lv}$, $E_{max,rv}$) also involve parasympathetic components. As illustrated in figure 1, neuronal activity (again described by sigmoid functions) in distinct regions of the nucleus tractus solitarius (NTS) process afferent inputs. Thus, the activity of a functional block, j, in the parasympathetic pathway is described generically as

$$f_{o,j} = S(f_{i,j}) = \frac{f_{min,j} + f_{max,j} e^{\frac{f_{i,j} - f_{mid,j}}{k_j}}}{1 + e^{\frac{f_{i,j} - f_{mid,j}}{k_j}}}. \quad (35)$$

where $f_{o,j}$ is the spike rate of signal output from block j, $f_{i,j}$ is the frequency of the input signal, and $f_{min,j}$, $f_{max,j}$, and $fmid, j$ are parameters (listed in Table 5) of the sigmoid.

Linear combinations of the output from the NTS serve as input to the nucleus ambiguus (NA) and the dorsal motor nucleus of the vagus (DMV) where parasympathetic

Table 5. Parameters of the sigmoid functions in the vagal pathway

Functional group	f_{min}	f_{max}	k	f_{mid}
NTS_{br}	0.3	21.5	2.14	44.3
NTS_{cpr}	0.451	28.357	21.636	10.2
NTS_{lsr}	2.75	31.57	27.52	10
NA	4.88	15.78	2.55	60
DMV	2.59	6.66	1.24	43.1
NA_{ctr}	0.61	11	1.2	9.8

contributions to heart period and ventricular elastances are regulated. A third neuronal group, $NActr$, is also introduced since the NA exerts a slight influence on contractility.

$$\begin{bmatrix} f_{i,NA} \\ f_{i,DMV} \end{bmatrix} = \begin{bmatrix} 1 & 1 & 1 \\ 0 & 1 & 1 \end{bmatrix} \begin{bmatrix} f_{o,NTS_{br}} \\ f_{o,NTS_{cpr}} \\ f_{o,NTS_{lsr}} \end{bmatrix} \tag{36}$$

$$f_{o,NA} = S_{NA}(f_{i,NA}) \tag{37}$$

$$f_{o,DMV} = S_{DMV}(f_{i,DMV}) + S_{NActr}(f_{o,NA}) \tag{38}$$

The vagal contribution to heart period is then described by

$$\sigma_{Tv} = G_{T,v} f_{NA}(t - D_{T,v}) \tag{39}$$

$$P_{corrected,i} = \frac{P_i - OverlapP_i}{RS_i} \tag{40}$$

$$Purity_i = \frac{P_{corrected,i}}{\Sigma P_{corrected,i}} \tag{41}$$

$$\dot{\Delta T_v} = \frac{1}{\tau_{T,v}} \left[-\Delta T_v + \sigma_{T,v}\right]. \tag{42}$$

The gain, $G_{T,v}$, latency, $D_{T,v}$ and time constant, $\tau_{T,v}$ are provided in Table 4. The vagal contribution to contractility (elastance) follows a similar pattern but is driven by $f_{o,DMV}$. Finally, the instantaneous heart period is sum of the sympathetic and vagal contributions.

$$T = \Delta T_s + \Delta T_v + T_0 \tag{43}$$

Modification 3: In order to capture the trademark 'irregularly irregular' heart period during AF, we describe the shortened intra-atrial activation times in the left atrium using a Normal distribution. The resulting distribution (not necessarily Gaussian) of intra-ventricular activation times or T, is a function of the stochastic atrial input and the refractory period of the atrioventricular node.

References

1. Park JH, Gorky J, Ogunnaike B, Vadigepalli R, Schwaber JS. Investigating the effects of brainstem neuronal adaptation on cardiovascular homeostasis. Frontiers in neuroscience. 2020;14:470.
